# Dietary patterns and knowledge-attitude-practice factors are associated with late recurrence of non-muscle-invasive bladder cancer: a case–control study

**DOI:** 10.3389/fnut.2026.1842495

**Published:** 2026-05-29

**Authors:** Qiquan Wu, Xiaodong Qing, Wenbo Gao

**Affiliations:** 1 Department of Urology, Ningbo Integrated Traditional Chinese and Western Medicine Hospital, Ningbo, Zhejiang, China; 2 Department of Surgery, Ningbo Integrated Traditional Chinese and Western Medicine Hospital, Ningbo, Zhejiang, China

**Keywords:** bladder cancer, cruciferous vegetables, diet, hydration, knowledge-attitude-practice, preserved seafood, recurrence

## Abstract

**Background:**

The influence of long-term dietary habits on bladder cancer (BC) recurrence beyond the first postoperative year remains unclear, particularly in populations with distinct dietary traditions. This study investigated the associations between dietary knowledge, attitudes, and practices (KAP) and late recurrence (≥12 months after transurethral resection of bladder tumor, TURBT) in a coastal Zhejiang population.

**Methods:**

This retrospective case–control study included 264 patients with non-muscle-invasive bladder cancer (NMIBC), comprising 85 cases with recurrence ≥12 months post-TURBT and 179 recurrence-free controls who were frequency-matched for age, sex, tumor stage, and follow-up duration. Tumor grade was not a matching criterion but was adjusted for in multivariable analyses. Dietary KAP was assessed using a structured questionnaire, and habitual dietary intake was evaluated using a culturally adapted food frequency questionnaire (FFQ). Multivariable logistic regression was used to identify independent dietary factors associated with recurrence.

**Results:**

Patients in the recurrence group had significantly lower dietary knowledge scores (5.9 vs. 7.3, *p* < 0.001) and less favorable attitudes (28.7 ± 5.8 vs. 32.4 ± 4.9, *p* = 0.003) than controls. High consumption of preserved seafood (≥1/week: aOR = 2.15, 95% CI: 1.28–3.62), low intake of cruciferous vegetables (<1 serving/week: aOR = 2.41, 95% CI: 1.42–4.10), and inadequate daily water intake (<1.0 L/day: aOR = 2.58, 95% CI: 1.38–4.82) were independently associated with increased recurrence risk, with significant dose–response trends (all *p*-for trend <0.05). Higher knowledge scores correlated significantly with adherence to protective dietary practices.

**Conclusion:**

In this coastal Zhejiang population, poor dietary knowledge, unfavorable attitudes, and specific dietary patterns- particularly high preserved seafood intake, low cruciferous vegetable consumption and inadequate hydration- were independently associated with late BC recurrence. These findings support incorporating culturally tailored dietary education into routine survivorship care for patients with BC.

## 1 Introduction

Bladder cancer (BC) is one of the most common malignancies of the urinary tract, with non-muscle-invasive bladder cancer (NMIBC) accounting for approximately 75% of new diagnoses (1). Although transurethral resection of bladder tumor (TURBT) represents the standard treatment, patients with NMIBC face a substantial risk of recurrence, necessitating lifelong surveillance (for example, cystoscopic examination) and repeated interventions, which impose a considerable clinical and economic burden for patients and their families (2). While established risk models such as the European Organization for Research and Treatment of Cancer (EORTC) tables could effectively predict early recurrence based on tumor-related factors, the determinants of late recurrence- particularly those beyond the first postoperative year- remain less well characterized (3).

Diet has been hypothesized to influence BC outcomes through a biologically plausible mechanism: many dietary metabolites and carcinogens are excreted in urine, bringing them into direct and prolonged contact with the bladder urothelium (4). Experimental and epidemiological studies have suggested that certain dietary factors might modulate risks of BC. For instance, high fluid intake may dilute urinary carcinogens, while certain fruits and vegetables, especially cruciferous vegetables, contain isothiocyanates with potential properties of chemoprevention (5, 6). Conversely, preserved or processed foods can be sources of N-nitroso compounds and heterocyclic amines, which are known urinary carcinogens (7). However, most available evidence has been derived from Western populations with distinct dietary traditions (8), and data regarding dietary patterns for recurrence in Asian populations- particularly those with unique regional habits such as coastal Zhejiang *population*- remain scarce.

Beyond actual dietary intake, cognitive and behavioral factors may also play critical roles in shaping long-term dietary habits. The Knowledge-Attitude-Practice (KAP) framework posits that health-related behaviors are driven by an individual’s knowledge and attitudes; and this model has been widely applied in chronic disease management and health education (9, 10). Nevertheless, its application to BC survivors, and specifically its association with recurrence outcomes, has not been systematically investigated.

Importantly, the timing of BC recurrence may reflect distinct underlying mechanisms. Most existing studies do not distinguish early from late recurrence, potentially obscuring the role of modifiable lifestyle factors. Early recurrence, typically within the first 12 months after TURBT, is largely driven by surgical factors, such as incomplete resection or residual disease (11). In contrast, recurrence occurring beyond the first postoperative year is more likely influenced by ongoing biological processes, including field cancerization effects and sustained exposure to environmental and lifestyle factors (12, 13). Therefore, the prognostic significance of a 12-month cutoff has been studied, and further validated in a recent study of intermediate-risk NMIBC (14), which demonstrated that patients with recurrence >12 months after diagnosis had significantly better progression-free survival (3-year PFS: 74%) compared with those recurring before 12 months (3-year PFS: 42%). These findings supported the use of a 12-month landmark to distinguish late recurrence, which might be more amenable to modification by sustained lifestyle factors, especially dietary habits. Focusing on late recurrence (12-month cutoff) thus provides a more appropriate framework to distinguish early recurrence, which is largely attributable to residual disease or surgical factors, from late recurrence, which is more likely influenced by sustained environmental and lifestyle exposures.

In the present study, we conducted a retrospective case–control study in a coastal Zhejiang population to compare dietary knowledge, attitudes, and long-term dietary patterns between NMIBC patients with and without recurrence occurring ≥12 months after TURBT. Zhejiang Province is a region representative of the dietary traditions typical of coastal Chinese communities. Common local preserved seafood items include “salted yellow croaker”, “dried salted shrimp”, and “fermented raw crab”. These items are traditionally preserved using high concentrations of sodium chloride, air-drying, or brine fermentation, resulting in high sodium and potential N-nitroso compound content. We thus aimed to identify dietary factors independently associated with late recurrence and to explore the relationship between cognitive factors and actual dietary behaviors, with the goal of guiding culturally tailored dietary guidance for BC survivorship care.

## 2 Methods

### 2.1 Study design and participants

This retrospective case–control study was conducted at a tertiary urological oncology center in eastern Zhejiang, China. Patients who underwent TURBT for histologically confirmed NMIBC (stages Ta, T1, or carcinoma *in situ* (CIS)) between January 2019 and December 2022 were identified from the electronic medical record system of the hospital. All eligible patients were routinely followed after surgery according to institutional surveillance protocols.

To minimize the influence of early recurrence related to residual tumor, surgical factors, or inherent tumor biology, only patients with recurrence occurring ≥12 months after the initial TURBT were included in the recurrence group (R Group). Patients who remained recurrence-free during follow-up period were included in the control group (C Group). To reduce potential misclassification due to insufficient observation time, controls were required to have a minimum follow-up duration of at least 30 months and were frequency-matched to cases on age (±5 years), sex, initial tumor stage, and follow-up duration to ensure comparable observation periods. Tumor grade (low-grade vs. high-grade) was not a matching criterion due to sample size constraints but was included as a covariate in all multivariable regression models. The matching ratio was approximately 2:1 (179 controls to 85 cases). For C Group, dietary exposure was anchored to a matched index date corresponding to the median recurrence time of the R Group, so as to improve temporal comparability. Clinical and demographic information was collected from medical records and supplemented by structured interviews at the time of questionnaire administration.

Intravesical therapy was administered according to institutional protocols and Chinese guidelines. Adjuvant intravesical chemotherapy (e.g., mitomycin C 40 mg or gemcitabine 1,000 mg weekly for 8 weeks) was generally recommended for intermediate-risk NMIBC, while BCG immunotherapy (81 mg weekly for 6 weeks, followed by maintenance within 12 months) was preferred for high-risk NMIBC. The proportion of patients receiving any intravesical therapy was compared between groups and included as a covariate in sensitivity analyses.

Inclusion and exclusion criteria of the participants were listed in Table 1.

**Table 1:** Study eligibility criteria.

Category	Inclusion criteria	Exclusion criteria
Recurrence group (R group)	1. Histologically confirmed recurrence of NMIBC (Ta/T1/CIS).	1. Recurrence within the first 12 months post-TURBT.
	2. Recurrence diagnosed ≥12 months after the initial TURBT.	2. Muscle-invasive bladder cancer (MIBC, stage ≥T2) at initial diagnosis.
	3. Age ≥ 50 years.	3. Presence of cognitive disorders or major illnesses impairing communication or recall.
	4. Clear consciousness, self-care ability, and willingness to provide informed consent.	4. Unwillingness to participate.
	5. Follow-up duration ≥30 months.	5. Concurrent or prior history of upper tract urothelial carcinoma.
Control group (C group)	1. No evidence of recurrence during follow-up.	1. Any history of bladder cancer recurrence.
	2. Age ≥ 50 years.	2. Muscle-invasive bladder cancer (MIBC, stage ≥T2) at initial diagnosis.
	3. Clear consciousness, self-care ability, and willingness to provide informed consent.	3. Presence of cognitive disorders or major illnesses impairing communication or recall.
	4. Follow-up duration ≥30 months.	4. Unwillingness to participate.
		5. Concurrent or prior history of upper tract urothelial carcinoma.

To avoid immortal time bias, for each case in the R Group (recurrence at time T), the matched control was assigned an “index date”- exactly T months after their own surgery. Controls were required to be recurrence-free up to this index date. This ensured that the observation period at risk was comparable between cases (surgery to recurrence) and controls (surgery to index date).

This study was approved by the Hospital Ethical Committee and conducted in accordance with the Declaration of Helsinki. Written informed consent was obtained from all participants.

### 2.2 Dietary KAP questionnaire

The Dietary KAP questionnaire was developed through a systematic review of the literature on diet and BC prognosis, including “Expert Consensus on Nutritional Management During the Rehabilitation Period of Cancer Patients (2022 Edition)” and “Chinese Guidelines on nutritional support in patients with tumor (2017 Edition),” in combination with relevant meta-analyses and pooled analyses (15–17). Its content was also aligned with general dietary recommendations for cancer survivors outlined in established clinical practice guidelines (18, 19). The initial draft was reviewed and modified by a panel of five experts (two urologists, two clinical nutritionists and one nursing expert). The feedback process allowed us to refine the questionnaire’s content. Content validity was ensured by expert panel review, and face validity was established through a pilot test with 30 BC survivors not included in the present study. None of them reported confusing items. After these clarifications, the face validity of the KAP questionnaire was confirmed. The final version demonstrated good internal consistency, with a Cronbach’s *α* coefficient of 0.910. Construct validity was not formally assessed (e.g., via factor analysis), which is acknowledged as a limitation.

The formal questionnaire was written in Chinese (an English version is provided in Supplementary material 1), and comprised four dimensions with a total of 36 items: demographic information (9 items), knowledge dimension (11 items), attitude dimension (8 items), and practice dimension (8 food categories). It was designed to be culturally relevant, particularly for the local (coastal Zhejiang) population. The demographic information included age, sex, cigarette smoking history, initial tumor pathology, and treatment details. The KAP dimensions included:

1. *Knowledge dimension*: It assessed participants’ understanding of diet-related factors for BC recurrence. These items consisted of 11 “true/false/do not-know” statements covering topics, such as the benefits of drinking water and green tea, cruciferous vegetables, as well as risks associated with processed meats, fried foods, and preserved seafood. Each correct response was scored 1 point, incorrect or “do not know” responses 0 point, yielding a total knowledge score ranging between 0 and 11.
2. *Attitude dimension*: Attitudes toward dietary modification were evaluated using 8 statements rated on a 5-point Likert scale from “strongly disagree” (1 point) to “strongly agree” (5 points). These items measured perceived importance of diet, self-efficacy, family support, perceived barriers, and willingness to adjust intake of specific foods, including local preserved seafood. The total attitude score ranged from 8 to 40.
3. *Practice dimension*: Dietary practices were assessed using a localized “Food Consumption Frequency Table” covering 8 core food/beverage categories selected for their direct relevance to BC prognosis and regional dietary patterns. These categories included: (a) daily water intake; (b) cruciferous vegetable consumption; (c) total fruit and vegetable intake; (d) red meat consumption; (e) preserved seafood intake (regionally specific); (f) fresh seafood consumption (with cooking methods); and (g) tea drinking (mainly green tea). Although processed meat is a recognized dietary risk factor (20), its regular consumption was reported to be relatively infrequent in our pilot survey of the local population (21). Moreover, its potential risk may overlap with that of red meat intake and high-temperature cooking methods (22), which were already assessed elsewhere in the questionnaire. Therefore, processed meat was not included as a separate item in the practice dimension; instead, knowledge regarding processed meats was evaluated in the Knowledge dimension (Item 2). This approach thus enabled an efficient assessment of key dietary behaviors within the KAP framework.

Although the practice dimension of the KAP questionnaire included several food-related behavior items, it was designed primarily to capture general dietary behavior patterns and adherence tendencies within the behavioral framework. To obtain a more comprehensive assessment of habitual dietary exposure, a localized food frequency questionnaire (FFQ) was additionally administered. The FFQ enabled a more detailed and semi-quantitative evaluation of habitual dietary intake for etiological analysis. The KAP instrument captured behavioral determinants, whereas the FFQ provided quantitative exposure assessment; together they enabled exploration of a cognition- behavior- outcome pathway.

### 2.3 Comprehensive dietary intake assessment

This localized FFQ was adapted from a previously validated Chinese instrument (23) to better reflect regional dietary patterns in coastal Zhejiang population. Modifications were made to enhance cultural relevance and exposure specificity. These included the addition of commonly consumed regional food items, detailed categorization of seafood (sea fish, freshwater fish, shrimp, crab, shellfish, and seaweed), and the incorporation of a structured module assessing cooking methods for meats and fish. Portion sizes were quantified using local household measures and standardized reference objects. The final questionnaire comprised more than 40 specific food items across 9 major food groups (grains, vegetables, fruits, seafood, meat, legumes, beverages, condiments, and other foods) (an English version is provided in Supplementary material 2). The FFQ was adapted from a previously validated instrument, but formal validation against biomarkers or dietary records in this specific population was not performed, which we acknowledge as a limitation.

This instrument assessed participants’ habitual consumption frequency over a standardized 12-month recall period. For the R Group, dietary intake referred to the 12 months preceding the diagnosis of recurrence, whereas for the C Group it referred to the 12 months preceding the interview. For each food item, portion sizes were specified using local household measures (24) (e.g., bowls, cups) in combination with standardized reference objects (such as common household items). Detailed verbal descriptions were provided by the interviewers. The data derived from this FFQ thus provided the basis for categorizing dietary intake into distinct consumption levels.

**Figure 1:**
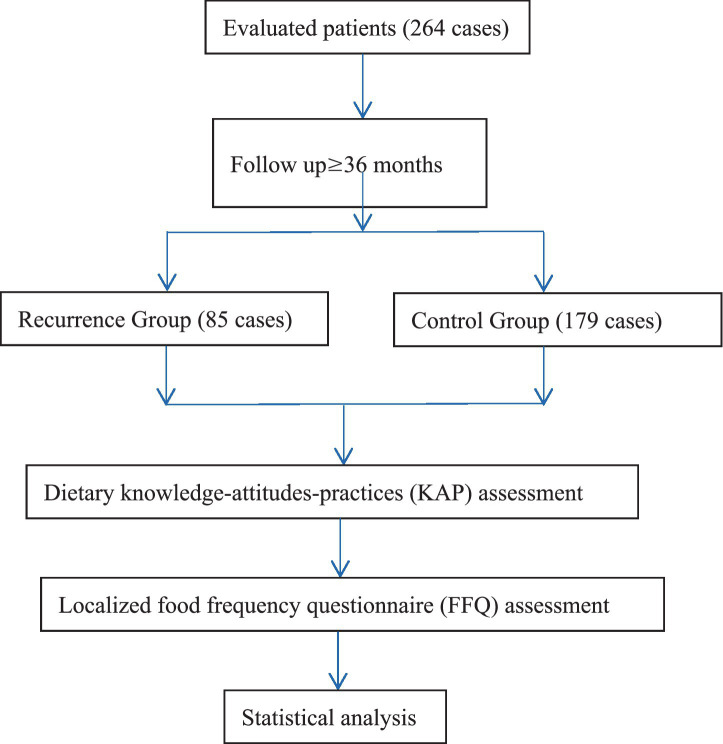
Study flow chart.

In this study, all data were collected through structured, face-to-face interviews conducted by two trained interviewers. To ensure the reliability of recall and minimize fatigue, the KAP questionnaire was administered first (taking approximately 10 min), followed by the comprehensive local FFQ (approximately 15 min). The study flow chart is seen in Figure 1.

### 2.4 Statistical analysis

Data were analyzed using SPSS version 20.0 (IBM, Armonk, NY, United States). Continuous variables were presented as mean ± standard deviation (SD) and compared using independent samples *t*-tests. Categorical variables were expressed as numbers (percentages) and compared using the Chi-square test or Fisher’s exact test as appropriate. Spearman’s rank correlation was used to assess the correlation between KAP scores and dietary intake frequencies.

Prior to multivariable analysis, multicollinearity among independent variables was assessed using variance inflation factors (VIFs); all VIFs were below 2.5, indicating no significant multicollinearity.

Multivariable logistic regression models were constructed a priori to evaluate independent associations between dietary factors and recurrence. Clinically relevant covariates, including age, sex, smoking status (current, former, never), educational level, BMI, clinical T stage, and follow-up duration, were simultaneously included regardless of univariate significance. Smoking status and educational level, which differed significantly between groups at baseline, were thus included as covariates in all multivariable models. Knowledge and attitude scores were analyzed both as continuous variables and as categorical variables (median split). For the KAP-practice concordance analysis, favorable intake levels for key dietary components were predefined according to existing literature (25, 26) and the categorical structure of the FFQ (e.g., preserved seafood <1/week, cruciferous vegetables ≥2/week, water intake ≥1.5 L/day). To formally test dose–response relationships, the Cochran-Armitage test for trend was used for categorical variables in logistic regression, with median values assigned to each exposure category. Adjusted odds ratios (ORs) with 95% confidence intervals (CIs) were reported. All tests were two-sided, and *p* < 0.05 was considered statistically significant.

## 3 Results

### 3.1 Participant characteristics

A total of 264 participants were included in the final analysis, comprising 85 patients in the R Group and 179 in the C Group. The matching achieved comparability between groups for age, sex, clinical T stage, and follow-up duration (all *p* > 0.05). All participants completed the questionnaires, with a response rate of 100%.

Baseline characteristics of the study population are summarized in Table 2. The mean age of participants was 68.2 ± 8.3 years, and 72.3% were male. The two groups were comparable with respect to age, sex, BMI, clinical T stage, and time since surgery (all p > 0.05). The median time from initial TURBT to recurrence diagnosis in the R Group was 18.5 months (IQR: 14.0–26.0 months).

**Table 2:** Baseline and surgical characteristics of the study groups.

Characteristic	C group (*n* = 179)	R group (*n* = 85)	*p*-value
Age (years), mean ± SD	68.5 ± 8.1	67.8 ± 8.7	0.521
Male, *n* (%)	131 (73.2)	60 (70.6)	0.892
BMI (kg/m^2^), mean ± SD	24.5 ± 3.9	25.1 ± 4.1	0.294
Educational degree, *n* (%)			0.015
Junior high school or below	58 (32.4)	47 (55.3)	
High school/vocational school	72 (40.2)	25 (29.4)	
College/University or above	49 (27.4)	13 (15.3)	
Smoking status, *n* (%)			<0.001
Current smoker	25 (14.0)	35 (41.2)	
Former smoker	34 (19.0)	6 (7.1)	
Never smoker	120 (67.0)	44 (51.8)	
Clinical T stage, *n* (%)			0.268
Ta	92 (51.4)	40 (47.1)	
T1	86 (48.0)	42 (49.4)	
CIS	1 (0.6)	3 (3.5)	
Received intravesical therapy, *n* (%)	104 (58.1)	53 (62.4)	0.512
Time since surgery (months), mean ± SD	44.2 ± 14.7	41.8 ± 13.6	0.092
Follow-up duration for C group (months), mean ± SD	44.2 ± 4.7	—	—
Time to recurrence for R group, median (IQR)	—	18.5 (14.0–26.0)	—

However, smoking status differed significantly between groups, with a higher proportion of current smokers in the R Group (41.2% vs. 14.0%, *p* < 0.001). Educational level also differed significantly between groups (*p* = 0.015).

### 3.2 Stratified analysis of dietary knowledge and attitudes

Dietary knowledge scores were significantly lower in R Group than in C Group (5.9 ± 2.4 vs. 7.3 ± 2.1, *p* < 0.001) (Table 3). Similar differences were observed across strata of smoking status and educational level.

**Table 3:** Stratified analysis of dietary knowledge and attitude scores.

Stratification variable	Group	Knowledge score (mean ± SD)	*p*-value	Attitude score (mean ± SD)	*p*-value
Overall	C group (*n* = 179)	7.3 ± 2.1	<0.001	32.4 ± 4.9	0.003
	R group (*n* = 85)	5.9 ± 2.4		28.7 ± 5.8	
By smoking status					
Ever-smokers (current + former)	C group (*n* = 59)	6.8 ± 2.3	0.002	31.5 ± 5.1	0.030
	R group (*n* = 41)	5.5 ± 2.5		28.1 ± 6.0	
Never-smokers	C group (*n* = 120)	7.6 ± 1.9	<0.001	32.8 ± 4.8	0.008
	R group (*n* = 44)	6.2 ± 2.3		29.2 ± 5.6	
By educational level					
Junior high or below	C group (*n* = 58)	6.2 ± 2.0	0.001	30.9 ± 5.3	0.041
	R group (*n* = 47)	4.8 ± 2.1		27.8 ± 6.2	
High school/vocational	C group (*n* = 72)	7.6 ± 2.0	0.013	32.8 ± 4.6	0.038
	R group (*n* = 25)	6.4 ± 2.2		29.5 ± 5.1	
College or above	C group (*n* = 49)	8.2 ± 1.8	0.185	33.8 ± 4.5	0.210
	R group (*n* = 13)	7.5 ± 2.0		31.6 ± 5.3	

*p*-values are for between-group comparisons (C group vs. R group) within each stratum, calculated using independent samples *t*-test.

As shown in the table, among ever-smokers, knowledge scores remained significantly lower in R Group than in C Group (*p* = 0.002). A comparable pattern was also observed among never-smokers (*p* < 0.001).

Stratification by educational level showed that knowledge scores were significantly lower in the R Group with junior high school education or below (*p* = 0.001) and those with high school or vocational education (*p* = 0.013), whereas no significant difference was observed among participants with college education or above (*p* = 0.185).

Attitude scores were also significantly lower in R Group compared with C Group (28.7 ± 5.8 vs. 32.4 ± 4.9, *p* = 0.003). Similar trends were also observed across smoking and educational strata (Table 3).

### 3.3 Dietary factors associated with late recurrence: multivariable-adjusted analysis

Analysis of the localized FFQ identified significant associations between several dietary factors and BC recurrence, as shown in Table 4.

**Table 4:** Associations between key dietary exposures and BC recurrence.

Variable	Category	C group *n* (%)	R group *n* (%)	Adjusted OR (95% CI)*	*p*-value	*p*-for trend
Preserved seafood	<1/month	98 (54.7)	35 (41.2)	1.00 (Ref)		0.008
	1–3/month	49 (27.4)	20 (23.5)	1.65 (0.92–2.96)	0.092	
	≥1/week	32 (17.9)	30 (35.3)	2.15 (1.28–3.62)	0.002	
Cruciferous vegetables	≥2/week	113 (63.1)	35 (41.2)	1.00 (Ref)		0.001
	1–<2/week	31 (17.3)	18 (21.2)	1.87 (1.05–3.33)	0.033	
	<1/week	35 (19.6)	32 (37.6)	2.41 (1.42–4.10)	0.001	
Daily water intake	1.5–2.0 L/day	75 (41.9)	28 (32.9)	1.00 (Ref)		0.003
	1.0–<1.5 L/day	41 (22.9)	25 (29.4)	1.92 (1.11–3.31)	0.019	
	<1.0 L/day	18 (10.1)	19 (22.4)	2.58 (1.38–4.82)	0.004	

*Adjusted for age, sex, smoking status, educational level, BMI, clinical T stage, and follow-up duration. Dietary variables were entered simultaneously in the fully adjusted model.

#### 3.3.1 Preserved seafood consumption

Weekly consumption (≥1 time/week) of preserved seafood was significantly more common in R Group than in C Group (35.3% vs. 17.9%, *p* = 0.002). Compared with participants consuming less than once per month, the adjusted OR for recurrence was 1.65 (95% CI: 0.92–2.96) for those consuming 1–3 times per month and 2.15 (95% CI: 1.28–3.62) for those consuming ≥1 time per week. The association reached statistical significance only for the highest intake category (≥1/week). A significant dose–response trend was observed across increasing intake categories (*p*-for trend = 0.008).

#### 3.3.2 Cruciferous vegetable intake

Frequent consumption of cruciferous vegetables was less common in R Group. While 63.1% of patients in C Group consumed cruciferous vegetables at least twice weekly, only 41.2% of cases in R Group reported this intake level (*p* = 0.001). When categorized into three intake levels (<1/week, 1-<2/week, ≥2/week), lower intake was associated with higher odds of recurrence, with the strongest association observed in the lowest intake category (OR = 2.41, 95% CI: 1.42–4.10). A significant protective dose–response trend was observed with increasing intake (*p*-for trend = 0.001).

#### 3.3.3 Daily water intake

Low daily fluid intake was more frequently reported in R Group. Consumption of less than 1.5 L/day was reported by 51.8% of recurrence cases compared with 33.0% of controls (*p* = 0.004). Higher water intake was associated with lower odds of recurrence across intake categories, with a significant dose–response trend (*p*-for trend = 0.003).

#### 3.3.4 Cooking methods for fresh fish

Total fresh fish intake did not differ significantly between groups. However, cooking methods varied. Participants in R Group reported higher frequencies of deep-fried (*p* = 0.013) and grilled fish (*p* = 0.042), whereas steaming and boiling were more commonly reported in C Group.

### 3.4 Comparison of other food groups based on the comprehensive FFQ

Habitual intake frequencies of other major food groups were compared between the two groups, to evaluate whether the observed dietary differences were specific for selected items (Table 5). Most food categories did not differ significantly, including staple grains, total vegetables (excluding cruciferous vegetables), fresh seafood, red meat, poultry, legumes, coffee, sugar-sweetened beverages, and salt use (all *p* > 0.05).

**Table 5:** Comparison of habitual intake frequencies for other major food groups between study groups.

Food group/item	Consumption frequency category	C group (*n* = 179) *n* (%)	R group (*n* = 85) *n* (%)	*p*-value
Staple grains (rice, noodles)	≥1 time/day	175 (97.8)	84 (98.8)	0.841
Total vegetables (excluding cruciferous)	≥1 serving/day	152 (84.9)	73 (85.9)	0.843
Total fruits	≥1 serving/day	102 (57.0)	44 (51.8)	0.046
Total fresh seafood	≥1 time/week	142 (79.3)	70 (82.4)	0.563
Red meat (pork)	≥1 time/week	121 (67.6)	62 (72.9)	0.389
Poultry (chicken, duck)	≥1 time/week	98 (54.7)	50 (58.8)	0.535
Legumes and products (Tofu, etc.)	≥1 time/week	135 (75.4)	67 (78.8)	0.549
Green tea	≥1 time/week	125 (69.8)	48 (56.5)	0.048
Coffee	≥1 time/month	20 (11.2)	7 (8.2)	0.462
Sugar-sweetened beverages	≥1 time/month	31 (17.3)	18 (21.2)	0.452
Condiment use (salt)^a^	High (>median)	92 (51.4)	50 (58.8)	0.259

Data are presented as number (percentage) of participants within each consumption frequency category. Although green tea and fruit consumption differed significantly between groups in univariate analysis, these associations were attenuated and became non-significant after multivariable adjustment (data not shown), suggesting potential confounding by other lifestyle or socioeconomic factors.

a High salt use was defined as reported monthly salt consumption above the median value of the entire cohort.

Of these items, statistically significant differences were observed for two dietary categories. Daily fruit consumption (≥1 serving/day) was reported by 57.0% of participants in C Group and 51.8% in R Group (*p* = 0.046). Green tea consumption (≥1 time/week) was also less frequent in R Group compared with C Group (56.5% vs. 69.8%, *p* = 0.048). However, it should be noted that, although statistically significant, the differences were relatively modest.

### 3.5 Association between dietary knowledge, attitudes, and predefined favorable dietary practices

As mentioned in the Statistical Analysis section, we further examined the relationship between dietary knowledge, attitudes, and adherence to predefined favorable dietary practices related to preserved seafood intake, cruciferous vegetable consumption, and daily water intake (Table 6).

**Table 6:** Association between dietary KAP scores and adherence to predefined favorable intake levels (derived from FFQ).

Recommended practice (from FFQ)	KAP metric	Group (by median split)	Adherent *n* (%)	Non-adherent *n* (%)	*p*-value (*χ*^2^ test)
Low preserved seafood intake (<1 serving/week)	Knowledge	High (score ≥7, *n* = 128)	100 (78.1)	28 (21.9)	<0.001
		Low (score <7, *n* = 136)	57 (41.7)	79 (58.3)	
	Attitude	High (score ≥31, *n* = 142)	101 (71.1)	41 (28.9)	<0.001
		Low (score <31, *n* = 122)	56 (45.9)	66 (54.1)	
Adequate water intake (≥1.5 L/day)	Knowledge	High (*n* = 128)	83 (65.2)	45 (35.8)	0.002
		Low (*n* = 136)	62 (45.8)	74 (54.2)	
	Attitude	High (*n* = 142)	93 (65.5)	49 (34.5)	0.010
		Low (*n* = 122)	52 (42.6)	70 (57.4)	
Frequent cruciferous vegetable intake (≥2 servings/week)	Knowledge	High (*n* = 128)	91 (71.6)	37 (28.4)	<0.001
		Low (*n* = 136)	66 (48.6)	70 (51.4)	
	Attitude	High (*n* = 142)	97 (68.3)	45 (31.7)	0.020
		Low (*n* = 122)	60 (49.2)	62 (50.8)	

As shown in the table, participants with higher dietary knowledge scores (≥median) demonstrated significantly greater adherence to favorable intake patterns across all three domains. Specifically, the proportion of participants consuming preserved seafood less than once per week was markedly higher in the high-knowledge group compared with the low-knowledge group (78.1% vs. 41.7%, *p* < 0.001). Similarly, adequate water intake (≥1.5 L/day) and frequent cruciferous vegetable consumption (≥2 servings/week) were significantly more prevalent among participants with higher knowledge levels.

A comparable, although slightly modest, pattern was observed for dietary attitudes. Participants with more positive attitudes toward dietary modification (scores ≥median) were more likely to report favorable intake behaviors, including lower preserved seafood consumption, higher cruciferous vegetable, and adequate daily fluid intake (all *p* < 0.05).

Correlation analyses further supported these findings. Knowledge scores were positively correlated with cruciferous vegetable intake frequency (*ρ* = 0.32, *p* < 0.001) and daily water intake (*ρ* = 0.25, *p* < 0.001), and negatively correlated with preserved seafood intake (*ρ* = −0.29, *p* < 0.001). Attitude scores demonstrated similar trend. These results suggested that dietary knowledge and attitudes were meaningfully aligned with actual dietary behaviors in this cohort.

## 4 Discussion

In this case–control study focusing on BC recurrence occurring ≥12 months after TURBT in a coastal Zhejiang population, we found that patients who remained recurrence-free had significantly higher dietary knowledge and attitude scores and were more likely to adhere to protective dietary practices. Specifically, frequent consumption of preserved seafood, low intake of cruciferous vegetables, and inadequate daily water intake were independently associated with increased risk of late recurrence. These associations remained significant after adjustment for major confounders, including smoking status. Moreover, our results supported the role of modifiable dietary behaviors in long-term oncologic outcomes.

Consistent with previous literature, current smoking was more prevalent in R Group than in C Group in our study. Smoking is a well-established risk factor for BC initiation and progression (27), and its higher frequency among recurrent patients underscores the importance of smoking cessation as a component of survivorship care. However, after multivariable adjustment that included smoking status, the associations between dietary factors and late recurrence remained significant, suggesting that the observed dietary effects were independent of smoking. This independence reinforces the notion that dietary modification represents a distinct and complementary strategy for secondary prevention beyond smoking cessation. Indeed, although smoking cessation remains the acknowledged lifestyle intervention for improving NMIBC outcomes, the potential role of dietary factors is increasingly recognized as an important and less explored issue for further investigation (26, 28).

**Figure 2:**
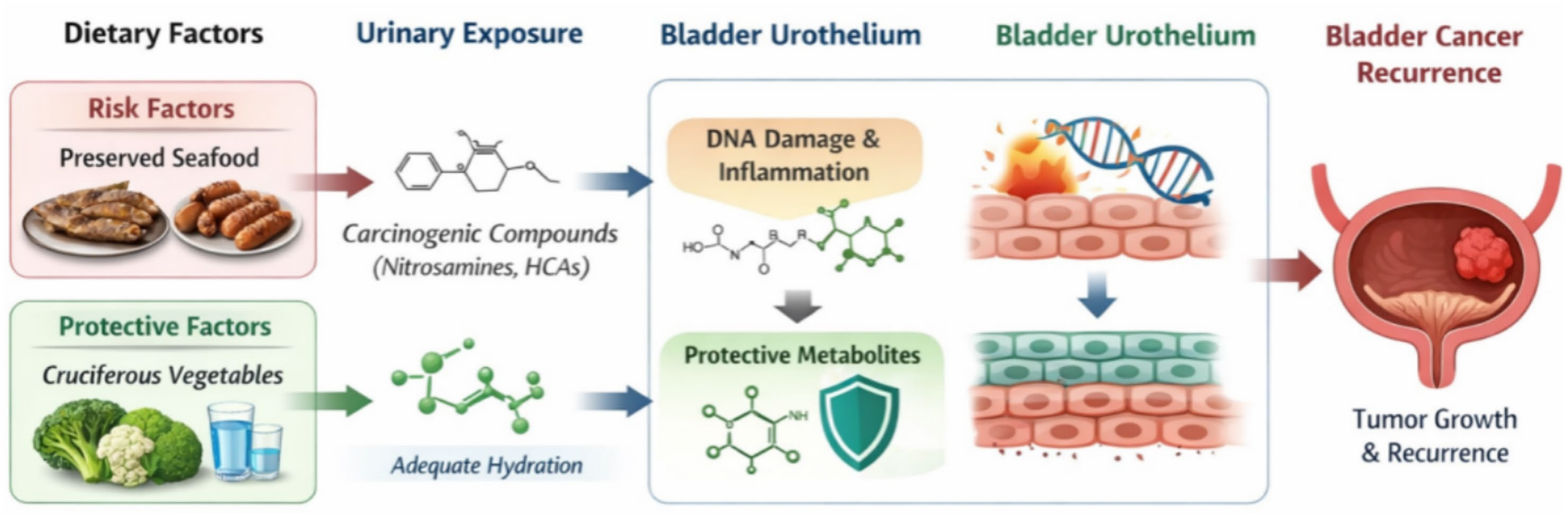
Schematic overview of proposed mechanisms linking dietary factors to bladder cancer recurrence.

The biological plausibility of our findings is supported by mechanistic pathways, as illustrated in Figure 2, which summarizes the proposed pathways through which these dietary factors may influence recurrence risk. While these mechanisms are well established in experimental models, it needs to be pointed out that our observational data provided real-world evidence linking these pathways to clinical outcomes in a high-risk population.

Our findings are consistent with several recent studies in Asian populations. A pooled analysis of Asian cohorts demonstrated that higher cruciferous vegetable intake was associated with reduced bladder cancer risk (29), supporting our observed inverse association. Regarding preserved foods, a meta-analysis including Asian populations found positive associations between salted fish consumption and risk of various cancers (30, 31), aligning with our regional observation. Furthermore, a prospective study from China reported that adequate hydration was associated with lower bladder cancer incidence (32, 33). These consistencies suggest that our findings may be generalizable to other Asian populations with similar dietary patterns, while also highlighting the unique regional relevance of preserved seafood exposure in coastal Zhejiang.

The positive association between preserved seafood consumption and BC recurrence is regionally relevant. Preserved seafood- such as salted fish and dried shrimp- is a traditional dietary staple in the area of coastal Zhejiang (8, 21). The long-term exposure of these products and subsequent carcinogenic compounds, particularly N-nitrosodimethylamine and other volatile N-nitrosamines which have been detected at notable levels in salted and dried seafood from the Zhejiang coastal region (34), may promote carcinogenesis through oxidative DNA damage and sustained epithelial irritation. The observation that patients more frequently consumed fried or grilled fish further supports the role of cooking methods in generating additional mutagens, including heterocyclic amines and polycyclic aromatic hydrocarbons (35), as they are formed during high-temperature cooking or processing and have been identified as dietary mutagens relevant to BC. However, it is important to note that the association for preserved seafood reached statistical significance only for weekly intake (OR = 2.15), while the estimate for monthly intake (OR = 1.65) had a confidence interval crossing 1.0, suggesting that the risk may become clinically meaningful only with higher exposure frequency. This finding aligns with broader evidence linking processed meat intake to increased bladder cancer risk, as demonstrated in a New England population (8).

In contrast, cruciferous vegetable intake exhibited a significant inverse association with BC recurrence. Cruciferous vegetables, such as broccoli and cabbage, are rich in glucosinolates, which are hydrolyzed to bioactive isothiocyanates (ITCs) such as sulforaphane (6). ITCs induce phase II detoxification enzymes (36), subsequently exert direct anticancer effects by modulating cell-cycle regulation and inducing apoptosis in human BC cells (37). Because urinary excretion concentrates ITC metabolites in the bladder, a localized chemopreventive effect is mechanistically supported. The observation of a protective effect in our study, even with modest intake (≥2 servings/week), suggested that sustained consumption of cruciferous vegetables might be a feasible strategy to modify long-term recurrence risk. Our findings are consistent with previous studies which showed that cruciferous vegetable consumption was related with improved BC survival (30) and reduced risk in pooled international analyses (29).

Adequate hydration is widely recognized as an independent protective factor, supporting the “dilution hypothesis.” (38) Higher fluid intake reduces urinary carcinogen concentration and contacting time with the bladder urothelium, thereby mitigating cumulative DNA damage (39). Given the direct and prolonged exposure of the bladder epithelium to urinary metabolites, even modest differences in fluid intake may have cumulative biological consequences, including sustained DNA damage and chronic inflammation, over extended periods. The simple behavioral intervention has consistent biological effect for primary and secondary prevention of BC (39). In our study, daily water intake ≥1.5 L/day was associated with significantly lower recurrence odds. In addition, hydration represents a low-cost, low-risk, and feasible intervention that could be readily incorporated into routine postoperative care.

A key contribution of this study is combining dietary assessment with the Knowledge-Attitude-Practice framework to show that better dietary knowledge and attitudes are linked to protective eating habits- such as limiting preserved seafood, eating cruciferous vegetables, and drinking enough water- which in turn are associated with lower late recurrence risk. These findings suggest that patient education can shape long-term behaviors and bring about better outcomes- reduction of BC recurrence. Clinically, this means that routine follow-up for NMIBC should go beyond cystoscopy and intravesical therapy to include structured dietary counseling. Specifically, assessing intake of regionally relevant foods like preserved seafood and providing culturally tailored dietary advice, rather than generic recommendations, may help identify at-risk patients and support sustainable lifestyle changes.

Several limitations should be acknowledged. First, the retrospective case–control design is inherently susceptible to bias, particularly recall bias. To minimize this, we used standardized interviewer-administered questionnaires with a defined 12-month recall period and structured probes. However, differential recall bias between cases and controls cannot be entirely excluded. Second, our definition requiring recurrence ≥12 months and follow-up ≥30 months deliberately excluded patients with early recurrence (<12 months), who constitute a substantial proportion of NMIBC recurrences in clinical context. This exclusion was intentional to isolate lifestyle-related late recurrences but limits generalizability to the broader NMIBC population, particularly those with more aggressive tumor biology. Third, the single-center design in a coastal Zhejiang population might limit the generalizability of this study to other regions with different dietary cultures. Fourth, the sample size, while adequate for primary analyses, precluded stratified analyses by tumor grade or intravesical therapy. Residual confounders from tumor grade and intravesical therapy cannot be excluded. Fifth, the localized FFQ, although culturally adapted and based on a previously validated instrument, has not been formally validated against common biomarkers in this population. Sixth, the absence of formal construct validity testing for the KAP questionnaire (e.g., via factor analysis) represents a methodological limitation. Furthermore, the KAP assessment was conducted at a single time point after recurrence (cases) or at enrollment (controls). We did not collect longitudinal KAP data during the first postoperative year, which limits our ability to assess dynamic changes in knowledge and attitudes or to establish temporal causality. Future prospective studies with serial KAP assessments, as well as randomized, multi-center trials are warranted to validate these findings and evaluate the efficacy of dietary interventions.

## 5 Conclusion

In this coastal Zhejiang population, patients who remained recurrence-free following TURBT demonstrated better dietary knowledge, more favorable attitudes, and healthier long-term eating habits in comparison to those who experienced recurrence beyond the first postoperative year. Frequent consumption of preserved seafood, low intake of cruciferous vegetables, and inadequate daily water intake were independently associated with an increased risk of late recurrence. These findings underscored the potential impact of modifiable postoperative dietary behaviors on long-term outcomes, and supported the routine integration of better dietary education and culturally tailored survivorship interventions into clinical practice. Further prospective studies and interventional trials are required to confirm these associations and evaluate the efficacy of targeted dietary strategies.

## Data Availability

The original contributions presented in the study are included in the article/Supplementary material, further inquiries can be directed to the corresponding author.
