# Supplementary material for: Dietary patterns and knowledge-attitude-practice factors are associated with late recurrence of non-muscle-invasive bladder cancer: a case–control study
Source: Front Nutr. 2026 May 29;13:1842495. doi: 10.3389/fnut.2026.1842495 (PMC13259901; doi:10.3389/fnut.2026.1842495)
Supplement: Supplementary file 1 [file Table_1.DOCX]

# Diet and Health Questionnaire for Postoperative Bladder Cancer Patients (Including Coastal Zhejiang Diet Assessment)

## Part 1: Basic Information

****1. Age:**** ______ years old

****2. Gender:****

Male :

Female:

****3. Are you a resident of coastal areas in eastern Zhejiang?****

Yes (please specify region: ____________)

No

****4. Highest Education Level:****

Primary school or below

Junior high school

High school/vocational school

College/University

Master's degree or above

****5. Current Occupation:**** ____________________

****6. Date of your first Transurethral Resection of Bladder Tumor (TURBT) surgery:**** ______ Year ______ Month

****7. Your smoking status:****

Never smoked

Quit smoking (quit for ______ years)

Currently smoking

****8. Your alcohol consumption:****

Never or rarely drink

Occasional social drinking

Frequent drinking

****9. (For recurrence group only) Date of your most recent recurrence diagnosis:**** ______ Year ______ Month

## Part 2: Dietary Knowledge (Please indicate if the following statements are true or false)

****1. Drinking plenty of water and urinating frequently helps reduce the risk of bladder cancer recurrence.****

True

False

Don't know

****2. High consumption of processed meats like bacon, ham, and sausage may increase recurrence risk.****

True

False

Don't know

****3. Eating more cruciferous vegetables like broccoli, cabbage, and white radish may be beneficial for preventing recurrence.****

True

False

Don't know

****4. As long as I adhere to intravesical therapy, I don't need to pay special attention to diet.****

True

False

Don't know

****5. Drinking tea (especially green tea) has potential protective effects on bladder health.****

True

False

Don't know

****6. To supplement nutrition, I should eat more red meat (pork, beef, lamb) after surgery.****

True

False

Don't know

****7. Eating adequate fruits and vegetables daily (e.g., 250g of fruit, 500g of vegetables) is part of a healthy diet.****

True

False

Don't know

****8. Frequent consumption of fried and grilled foods has no impact on bladder health.****

True

False

Don't know

****9. Preserved seafood commonly eaten in Zhejiang region, such as salted fish and salted shrimp, may increase the risk of bladder cancer recurrence.****

True

False

Don't know

****10. Moderate consumption of fresh fish (such as hairtail, yellow croaker) prepared by steaming or boiling is beneficial for bladder health.****

True

False

Don't know

****11. Frequently cooking fish by deep-frying or grilling may produce substances harmful to the bladder.****

True

False

Don't know

## Part 3: Dietary Attitudes and Beliefs

****Please read the following statements and check (√) the box that best represents your level of agreement.****

| **No.** | **Statement** | **Strongly Disagree** | **Disagree** | **Neutral** | **Agree** | **Strongly Agree** |
| --- | --- | --- | --- | --- | --- | --- |
| A1 | I believe adjusting dietary habits is very important for preventing bladder cancer recurrence. | [ ] | [ ] | [ ] | [ ] | [ ] |
| A2 | I am confident that I can change my unhealthy eating habits. | [ ] | [ ] | [ ] | [ ] | [ ] |
| A3 | Knowledge about the relationship between diet and bladder cancer confuses me. | [ ] | [ ] | [ ] | [ ] | [ ] |
| A4 | My family supports me in adjusting my diet for health reasons. | [ ] | [ ] | [ ] | [ ] | [ ] |
| A5 | Preparing healthy meals takes too much time and effort for me. | [ ] | [ ] | [ ] | [ ] | [ ] |
| A6 | I am willing to reduce my consumption of grilled and fried foods to lower recurrence risk. | [ ] | [ ] | [ ] | [ ] | [ ] |
| A7 | I will carefully follow dietary advice given by doctors or nutritionists. | [ ] | [ ] | [ ] | [ ] | [ ] |
| A8 | I understand the potential impact of Zhejiang's local preserved seafood (e.g., salted fish, marinated crab) on bladder health and am willing to adjust my consumption habits. | [ ] | [ ] | [ ] | [ ] | [ ] |

## Part 4: Retrospective Dietary Habit Survey (Food Frequency Questionnaire - FFQ)

****Core Instructions (Investigator must clearly inform):****

****(To case group)**** Now, please carefully recall your usual dietary habits during the ****approximately 1-year period before your last recurrence****.

****(To control group)**** Now, please carefully recall your usual dietary habits during the ****past 1 year****.

For each food category, select the ****consumption frequency**** that best matches your situation at that time.

### Food Frequency Questionnaire

| **Food Category and Common Examples** | **Average Consumption Frequency (Select one)** |
| --- | --- |
| ****1. Drinking water (including boiled water, purified water, mineral water)**** | [ ] Almost never [ ] Less than 3 cups per day (<750ml) [ ] 3-5 cups per day (750-1250ml) [ ] 6-8 cups per day (1500-2000ml) [ ] More than 8 cups per day (>2000ml) |
| ****2. Vegetables (all types, e.g., leafy greens, squash, tomatoes)**** | [ ] Almost never [ ] Less than 3 times per week [ ] 3-5 times per week [ ] Once per day [ ] Twice or more per day |
| ****3. Cruciferous vegetables (e.g., broccoli, cauliflower, cabbage, white radish)**** | [ ] Almost never [ ] 1-3 times per month [ ] Once per week [ ] 2-3 times per week [ ] 4 times or more per week |
| ****4. Fruits (all types)**** | [ ] Almost never [ ] Less than 3 times per week [ ] 3-5 times per week [ ] Once per day [ ] Twice or more per day |
| ****5. Red meat (pork, beef, lamb dishes, e.g., braised pork, stir-fried shredded meat)**** | [ ] Almost never [ ] 1-3 times per month [ ] 1-2 times per week [ ] 3-4 times per week [ ] 5 times or more per week |
| ****6. Preserved seafood (e.g., salted fish, salted shrimp, marinated crab, pickled mud snails)**** Note: These are characteristic foods of coastal Zhejiang region | [ ] Almost never [ ] Less than once per month [ ] 1-3 times per month [ ] 1-2 times per week [ ] 3 times or more per week |
| ****7. Fresh seafood (fish, shrimp, shellfish, etc., non-preserved)**** ****Main cooking method (multiple choices allowed):**** [ ] Steaming/Boiling [ ] Braised [ ] Deep-frying [ ] Grilling | [ ] Almost never [ ] Less than once per week [ ] 1-2 times per week [ ] 3-4 times per week [ ] 5 times or more per week |
| ****8. Tea (mainly green tea)**** | [ ] Never drink [ ] Occasionally drink (a few times per month) [ ] Often drink (a few times per week) [ ] Drink daily (<3 cups) [ ] Drink daily (≥3 cups) |

## End of Questionnaire

****End of questionnaire. Thank you again for your participation!****

****Questionnaire ID:**** ______

****Date of survey: / /****

****Group:****

Case group (recurred)

Control group (non-recurred)

****Investigator notes:**** ________________________________________________________________
