# Supplementary material for: Dietary patterns and knowledge-attitude-practice factors are associated with late recurrence of non-muscle-invasive bladder cancer: a case–control study
Source: Front Nutr. 2026 May 29;13:1842495. doi: 10.3389/fnut.2026.1842495 (PMC13259901; doi:10.3389/fnut.2026.1842495)
Supplement: Supplementary file 2 [file Table_2.DOCX]

### ****Food Frequency Questionnaire (FFQ) – Adapted for Eastern Coastal Ningbo Population****

#### ****I. Basic Information****

| **Item** | **Content** |
| --- | --- |
| Name |  |
| Gender | □Male □Female |
| Date of Birth | Year Month Day |
| Contact Information |  |
| Residential Area | □Urban □Township □Coastal Village |

#### ****II. Food Frequency Questionnaire (FFQ)****

##### ****(1) Grains and Products****

| **Food Item** | **Never** | **1–3 times/month** | **1–3 times/week** | **4–6 times/week** | **Once daily** | **Twice+ daily** |
| --- | --- | --- | --- | --- | --- | --- |
| Rice | □ | □ | □ | □ | □ | □ |
| Noodles | □ | □ | □ | □ | □ | □ |
| Congee | □ | □ | □ | □ | □ | □ |
| Bread/Cookies | □ | □ | □ | □ | □ | □ |

##### ****(2) Vegetables****

| **Food Item** | **Never** | **1–3 times/month** | **1–3 times/week** | **4–6 times/week** | **Once daily** | **Twice+ daily** |
| --- | --- | --- | --- | --- | --- | --- |
| Leafy greens | □ | □ | □ | □ | □ | □ |
| Cruciferous vegetables | □ | □ | □ | □ | □ | □ |
| Tomatoes/Cucumbers | □ | □ | □ | □ | □ | □ |
| Pickled vegetables | □ | □ | □ | □ | □ | □ |

##### ****(3) Fruits****

| **Food Item** | **Never** | **1–3 times/month** | **1–3 times/week** | **4–6 times/week** | **Once daily** | **Twice+ daily** |
| --- | --- | --- | --- | --- | --- | --- |
| Citrus fruits | □ | □ | □ | □ | □ | □ |
| Apples/Pears | □ | □ | □ | □ | □ | □ |
| Bananas | □ | □ | □ | □ | □ | □ |
| Local seasonal fruits | □ | □ | □ | □ | □ | □ |

##### ****(4) Seafood****

| **Food Item** | **Never** | **1–3 times/month** | **1–3 times/week** | **4–6 times/week** | **Once daily** | **Twice+ daily** |
| --- | --- | --- | --- | --- | --- | --- |
| Sea fish (e.g., hairtail, yellow croaker) | □ | □ | □ | □ | □ | □ |
| Freshwater fish | □ | □ | □ | □ | □ | □ |
| Shrimp | □ | □ | □ | □ | □ | □ |
| Crab | □ | □ | □ | □ | □ | □ |
| Shellfish (clams, razor clams) | □ | □ | □ | □ | □ | □ |
| Seaweed/Laver | □ | □ | □ | □ | □ | □ |
| Salted fish | □ | □ | □ | □ | □ | □ |

##### ****(5) Meat and Poultry****

| **Food Item** | **Never** | **1–3 times/month** | **1–3 times/week** | **4–6 times/week** | **Once daily** | **Twice+ daily** |
| --- | --- | --- | --- | --- | --- | --- |
| Pork | □ | □ | □ | □ | □ | □ |
| Chicken | □ | □ | □ | □ | □ | □ |
| Duck | □ | □ | □ | □ | □ | □ |
| Preserved meat (ham, cured meat) | □ | □ | □ | □ | □ | □ |

##### ****(6) Legumes and Products****

| **Food Item** | **Never** | **1–3 times/month** | **1–3 times/week** | **4–6 times/week** | **Once daily** | **Twice+ daily** |
| --- | --- | --- | --- | --- | --- | --- |
| Tofu | □ | □ | □ | □ | □ | □ |
| Soy milk | □ | □ | □ | □ | □ | □ |
| Dried tofu | □ | □ | □ | □ | □ | □ |

##### ****(7) Beverages****

| **Beverage** | **Never** | **1–3 times/month** | **1–3 times/week** | **4–6 times/week** | **Once daily** | **Twice+ daily** |
| --- | --- | --- | --- | --- | --- | --- |
| Green tea | □ | □ | □ | □ | □ | □ |
| Coffee | □ | □ | □ | □ | □ | □ |
| Sugar-sweetened beverages | □ | □ | □ | □ | □ | □ |
| Seafood soup | □ | □ | □ | □ | □ | □ |

##### ****(8) Condiments (Monthly Consumption)****

| **Condiment** | **Amount** |
| --- | --- |
| Salt | g/month |
| Soy sauce | mL/month |
| Fish sauce | mL/month |
| Shrimp paste | g/month |
| Cooking oil | mL/month |

##### ****(9) Other Foods****

| **Food Item** | **Never** | **1–3 times/month** | **1–3 times/week** | **4–6 times/week** | **Once daily** | **Twice+ daily** |
| --- | --- | --- | --- | --- | --- | --- |
| Salted eggs | □ | □ | □ | □ | □ | □ |
| Preserved seafood | □ | □ | □ | □ | □ | □ |
| Fried foods | □ | □ | □ | □ | □ | □ |

| **Item** | **Options** |
| --- | --- |
| Taste preference | □Salty □Moderate □Light □Oily □Moderate □Light |
| Frequent consumption of preserved foods | □Yes □No |
| Frequent consumption of seafood soup | □Yes □No |
| Eating out frequency | □Almost daily □3–5 times/week □1–2 times/week □Several times/month □Rarely or never |
| Daily water intake | ______ mL/day |

#### ****III. Dietary Habits****

#### ****IV. Supplement Use****

| **Item** | **Response** |
| --- | --- |
| Do you take dietary supplements? | □Yes □No |
| If yes, please specify: |  |
